# Outcomes and outcomes measurements used in intervention studies of pelvic girdle pain and lumbopelvic pain: a systematic review

**DOI:** 10.1186/s12998-019-0279-2

**Published:** 2019-11-05

**Authors:** Francesca Wuytack, Maggie O’Donovan

**Affiliations:** 1 School of Nursing & Midwifery, Trinity College Dublin, 24 D’Olier Street, Dublin 2, Ireland; 2 School of Medicine, Discipline of Physiotherapy, Trinity College Dublin, Trinity Centre for Health Sciences, St James’s Hospital, Dublin 8, Ireland

**Keywords:** Pelvic girdle pain, Lumbopelvic pain, Outcomes, Outcome measurement, Systematic review

## Abstract

**Background:**

Pelvic girdle pain is a common problem during pregnancy and postpartum with significant personal and societal impact and costs. Studies examining the effectiveness of interventions for pelvic girdle pain measure different outcomes, making it difficult to pool data in meta-analysis in a meaningful and interpretable way to increase the certainty of effect measures. A consensus-based core outcome set for pelvic girdle pain can address this issue. As a first step in developing a core outcome set, it is essential to systematically examine the outcomes measured in existing studies.

**Objective:**

The objective of this systematic review was to identify, examine and compare what outcomes are measured and reported, and how outcomes are measured, in intervention studies and systematic reviews of interventions for pelvic girdle pain and for lumbopelvic pain (which includes pelvic girdle pain).

**Methods:**

We searched PubMed, Cochrane Library, PEDro and Embase from inception to the 11th May 2018. Two reviewers independently selected studies by title/abstract and by full text screening. Disagreement was resolved through discussion. Outcomes reported and their outcome measurement instruments were extracted and recorded by two reviewers independently. We assessed the quality of reporting with two independent reviewers. The outcomes were grouped into core domains using the OMERACT filter 2.0 framework.

**Results:**

A total of 107 studies were included, including 33 studies on pelvic girdle pain and 74 studies on lumbopelvic pain. Forty-six outcomes were reported across all studies, with the highest amount (26/46) in the ‘life impact’ domain. ‘Pain’ was the most commonly reported outcome in both pelvic girdle pain and lumbopelvic pain studies. Studies used different instruments to measure the same outcomes, particularly for the outcomes pain, function, disability and quality of life.

**Conclusions:**

A wide variety of outcomes and outcome measurements are used in studies on pelvic girdle pain and lumbopelvic pain. The findings of this review will be included in a Delphi survey to reach consensus on a pelvic girdle pain - core outcome set. This core outcome set will allow for more effective comparison between future studies on pelvic girdle pain, allowing for more effective translation of findings to clinical practice.

**Supplementary information:**

**Supplementary information** accompanies this paper at 10.1186/s12998-019-0279-2.

## Resumen en Español

**Introducción:**

El dolor de la cintura pélvica es un problema común durante el embarazo y el posparto con un impacto personal y social significativo. Los estudios que examinan la efectividad de intervenciones para el dolor de la cintura pélvica miden diferentes resultados, lo que dificulta el agrupamiento de los datos en un metanálisis para aumentar la certeza de las medidas del efecto. Un conjunto de resultados principales basado en un consenso puede abordar este problema. Primero, para desarrollar un conjunto de resultados principales, es esencial examinar sistemáticamente los resultados utilizados en los estudios existentes.

**Objetivo:**

El objetivo de esta revisión sistemática fue identificar, examinar y comparar qué resultados se miden y reportan, y cómo se los miden, en estudios de intervención y revisiones sistemáticas de intervenciones para el dolor de la cintura pélvica y para el dolor lumbopélvico.

**Método:**

Se realizaron búsquedas en PubMed, Cochrane Library, PEDro y Embase desde el inicio hasta el 11 mayo 2018. Dos revisores seleccionaron independientemente los estudios por título/resumen y texto completo. El desacuerdo se resolvió por discusión. Los resultados reportados y sus instrumentos de medición fueron extraídos por dos revisores independiente. Se evaluó la calidad de informe con dos revisores independientes. Los resultados se agruparon en dominios principales utilizando el filtro OMERACT 2.0.

**Resultados:**

Se incluyeron 107 artículos: 33 artículos sobre el dolor de la cintura pélvica y 74 artículos sobre el dolor lumbopélvico. Se informaron 46 resultados, principalmente (26/46) en el dominio “Impacto en la vida”. “El Dolor” fue el resultado más frecuente. Los estudios utilizaron diferentes instrumentos para medir los mismos resultados, particularmente para los resultados dolor, función, discapacidad y calidad de vida.

**Conclusiones:**

Se utiliza una amplia variedad de resultados y mediciones de resultados en estudios sobre el dolor de la cintura pélvica y el dolor lumbopélvico. Los resultados de esta revisión se incluirán en una encuesta Delphi, obtener para llegar a un consenso sobre un conjunto de resultados principales. Este conjunto de resultados principales permitirá una comparación más efectiva entre estudios sobre el dolor de la cintura pélvica, lo que permitirá un análisis más efectivo en la práctica clínica.

## Background

Pelvic Girdle Pain (PGP) has been defined as “pain between the posterior iliac crest and the gluteal fold, particularly in the vicinity of the sacroiliac joints, and pain may radiate to the posterior thigh and can also occur in conjunction with/or separately in the symphysis” [1] (pp797). In the past, it has sometimes been considered a subgroup of low back pain (LBP); however, PGP includes also pain at the pubic symphysis and is therefore considered a different entity. The term lumbopelvic pain (LPP) is a broader term that has been used to describe LBP and/or PGP without differentiation between the two groups [2].

Pelvic Girdle Pain is a common complaint during pregnancy, affecting 23 to 65% of women depending on how it is measured and defined [3, 4]. Although many women recover after the birth, 17% have continuing symptoms 3 months postpartum [2] and 8.5% have not recovered 2 years postpartum [5]. In Sweden, in a cohort of 371 women with PGP, 10% of women still had symptoms 11 years after the birth [6]. In another Swedish cohort, 40.3% had long term pain in the low back or pelvic girdle area 12 years postpartum [7]. Additionally, PGP is one of the leading causes of sick leave during pregnancy [8–10], resulting in large economic costs to families and society.

Studies examining the effectiveness of interventions for PGP measure different outcomes, making it difficult and sometimes impossible to pool data in meta-analysis to increase the certainty of effect measures [11, 12]. To address this issue, an international consensus-based Core Outcome Set (COS) for PGP is being developed (registration: http://www.comet-initiative.org/studies/details/958) [13]. The systematic review presented here forms the first key part of the PGP-COS (Pelvic Girdle Pain – Core Outcome Set) study and provides a structured overview of the outcomes and outcome measurements that are used across PGP as well as LPP (since this includes PGP) intervention studies and systematic reviews. It will feed into the larger PGP-COS study by providing a preliminary list of outcomes that will be included into an online Delphi survey and face-to-face consensus meeting to identify a final COS for PGP.

The objective of this systematic review was to identify and examine what outcomes are measured and reported, and how outcomes are measured, in intervention studies and systematic reviews of interventions for PGP.

## Methods

The protocol for this systematic review was published as part of the PGP-COS study protocol [13]. Criteria for considering papers for inclusion in the systematic review are outlined in Table 1. A second objective (To compare outcomes measured in intervention studies and systematic reviews on PGP to outcomes measured in studies on LPP) was added post hoc, since many studies that we identified in preliminary searches did not differentiate between LBP or PGP, and it was considered important to compare outcomes measured in these studies since LPP includes PGP. We analysed and have presented the results by the subgroups PGP and LPP.

**Table 1:** Inclusion criteria

Population	Women with PGP during or after pregnancy. PGP is defined as pain between the posterior iliac crest and the inferior gluteal fold, particularly in the vicinity of the sacroiliac joints, that may radiate in the posterior thigh and can occur in conjunction with or separately in the symphysis pubis [1]. Studies that examined a population of women with PGP resulting from specific pathologies (e.g. infection, spondyloarthropathies and trauma) were excluded.
Intervention	Any intervention (pharmacological or non-pharmacological) aimed to treat/prevent PGP.
Comparator	Any comparator intervention or control.
Outcome	Any outcome measured to assess/monitor PGP.
Study design	Intervention studies (randomised or non-randomised), systematic reviews of interventions.

### Search methods & study selection

The following databases were searched on the 11th May 2018 (from inception): PubMed, the Cochrane Library, PEDro and Embase. Details of search terms used for each database can be found in Additional file 1. No language or time filters were applied. We screened reference lists of included studies for further relevant studies. Citations were exported to Endnote and duplicates were removed. Two review authors (FW, MO) reviewed each citation independently against the inclusion criteria in two stages: (a) title and abstract screening and (b) full text screening, using Covidence software [14]. Disagreement was resolved through discussion.

### Data collection and synthesis

All outcomes (and their verbatim definitions) examined in the included studies were extracted by two reviewers (FW, MO) independently and their corresponding outcome measurement instruments/methods, where reported, were also recorded. The quality of outcome reporting was assessed using the six questions proposed by Harmen et al. [15] and this was conducted by two independent reviewers (FW, MO). The outcomes were then grouped into core outcome domains using the OMERACT (Outcome measures in rheumatology) filter 2.0 framework: (a) life impact; (b) resource use/economic impact; (c) pathophysiological manifestations and (d) death [16]. This framework aims to provide a structure for measuring outcomes and developing core outcome sets. Within the OMERACT framework ‘adverse events’ should also be flagged alongside the core domains. We therefore grouped adverse events into a separate domain [16]. The findings are synthesised and reported by these core domains, for PGP and LPP separately, for comparison. We have reported this systematic review according to the PRISMA guideline [17].

## Results

### Screening and selection of included papers

**Fig. 1:**
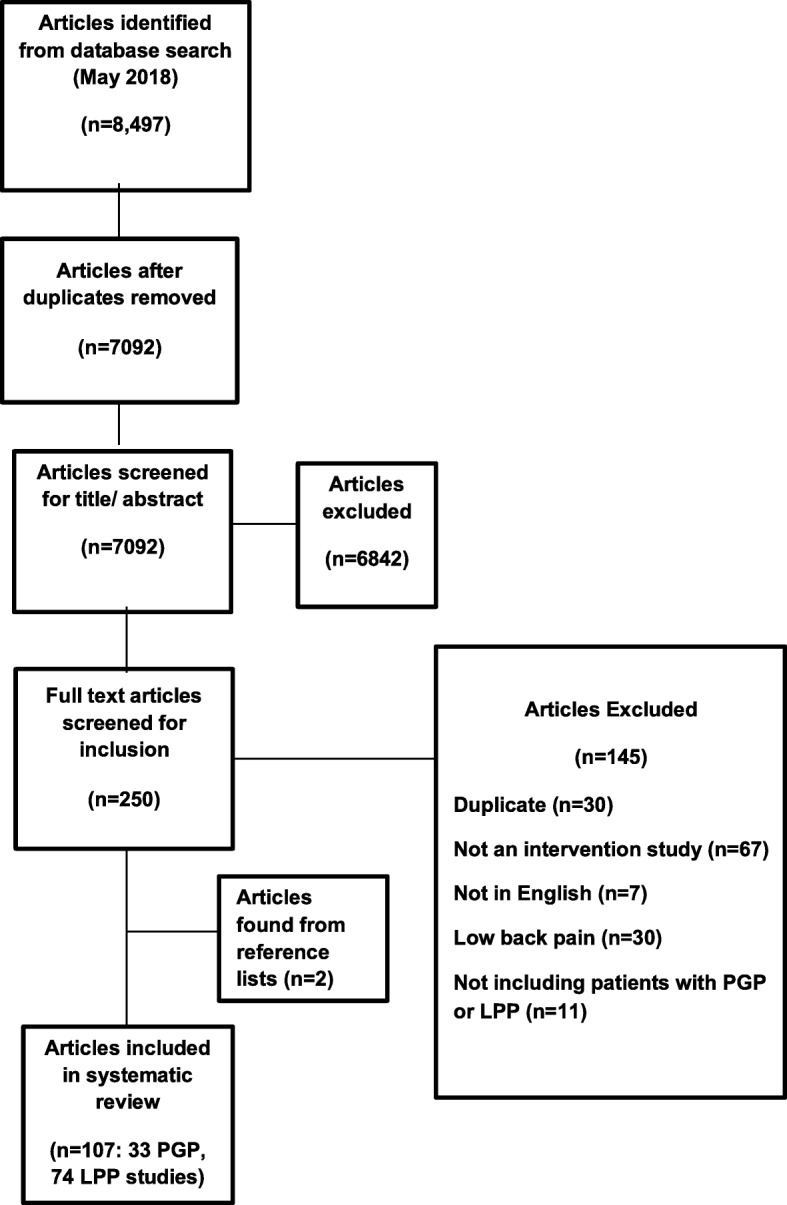
Screening and selection process of articles

A total of 7092 studies were identified from the initial search after removal of duplicates. We excluded 6842 studies during title and abstract screening, and the full texts of 250 articles were reviewed. A total of 145 studies were excluded at full text selection. Reasons for exclusion were: duplicates (*n* = 30), the wrong study design (*n* = 67), published in a language other than English (*n* = 7), examining LBP only (*n* = 30), or the wrong study population (*n* = 11). A further two studies were identified for inclusion from screening reference lists of included studies, with a total of 107 studies being included in the analysis. Figure 1 provides a flow diagram detailing the results of the search and selection process.

### Characteristics of included studies

Of the 107 studies included in the review, 31 were systematic reviews, 61 were Randomised Controlled Trials (RCTs) (including one follow up study [18] of another included study [19]), 11 were non-controlled intervention studies, two studies were non-randomised controlled studies [20, 21] and one study identified itself as a quasi-randomised design because no blinding of participates took place [22]. A total of 33 studies on PGP and 74 studies on LPP were included. Studies were published between the year 1991 and 2018, with 54% of studies published in the last 5 years. Studies were undertaken in a variety of geographical locations, across Europe, North and South America, Australia, New Zealand, Asia and Africa, with the highest percentage in Europe (66%), particularly in Sweden and Norway (30% of all included studies). Of the studies that focused on PGP, 24 studies (72.7%) included a physical examination as a requirement for the diagnosis of PGP. In comparison, only 18 (24.3%) of the studies focusing on LPP included a physical examination as a requirement for a diagnosis of LPP. Additional details of the characteristics of included studies can be found in Additional file 2.

An overview of the quality of reporting of the included studies [15] is presented in Tables 2 and 3, with higher quality reporting indicated by a yes vote, where applicable. All PGP studies (100%) and most LPP studies (94%) clearly reported and defined the primary outcome(s). About two thirds of studies did not differentiate between primary and secondary outcomes, making questions three and four not applicable. For transparency, the full quality of reporting assessment of each study determined by the six questions outlined by Harmen et al. [15] can be found in Additional file 3.

**Table 2:** Quality of reporting of included studies on PGP

Reporting Quality Question (PGP studies; *n* = 33)	Yes (%)	No (%)	N/A (%)
Q.1. Is the primary outcome clearly stated?	33 (100%)	0	0
Q.2. Is the primary outcome clearly defined so that another researcher would be able to reproduce its measurement?	32 (97%)	1 (3.0%)	0
Q.3. Are the secondary outcome clearly stated?	11 (33.3%)	1 (3.0%)	21 (63.6%)
Q.4. Are the secondary outcomes clearly defined?	11 (33.3%)	1 (3.0%)	21 (63.6%)
Q.5. Do the authors explain the use of the outcomes they have selected?	23 (69.7%)	10 (30.3%)	0
Q.6. Are methods used to enhance the quality of outcome measurement (e.g. repeated measurement, training), if appropriate?	27 (81.8%)	3 (9.1%)	3 (9.1%)

**Table 3:** Quality of reporting of included studies on LPP

Reporting Quality Question (LPP studies; *n* = 74)	Yes (%)	No (%)	N/A (%)
Q.1. Is the primary outcome clearly stated?	69 (93%)	3 (4%)	2 (2.7%)
Q.2. Is the primary outcome clearly defined so that another researcher would be able to reproduce its measurement?	62 (84%)	7 (9.5%)	5 (6.8%)
Q.3. Are the secondary outcome clearly stated?	25 (34%)	3 (4%)	46 (62%)
Q.4. Are the secondary outcomes clearly defined?	23 (31%	3 (4%)	48 (65%)
Q.5. Do the authors explain the use of the outcomes they have selected?	63 (85%)	10 (13.5%)	1 (1.4%)
Q.6. Are methods used to enhance the quality of outcome measurement (e.g. repeated measurement, training), if appropriate?	44 (59.5%)	7 (9.5%)	23 (31%)

### Outcomes and outcome measurements

A total of 46 outcomes were identified and categorised into core domains using the OMERACT filter 2.0 framework: ‘life impact’, ‘resource use/economic impact’, ‘pathophysiological manifestations’ and ‘death’. No outcomes were identified in the core domain ‘death’, but ‘adverse events’ outcomes were identified. Outcomes and their corresponding outcome measurements are presented separately for studies that focused on PGP or focused on LPP in the Tables 4, 5, 6 and 7. Of the 46 outcomes identified, 26 were in the life impact core domain (Table 4), five were in the resource-use/economic impact domain (Table 5), 11 were in the pathophysiological domain (Table 6), and four outcomes were classified in the adverse events domain (Table 7).

**Table 4:** Outcomes and outcome measurements identified in the ‘Life impact’ core domain for PGP and LPP respectively

Life Impact	PGP	LPP
Pain-related outcomes		
Pain Intensity	VAS [23–46] NRS [47–51] Not specified [52, 53] PGQ [20]	VAS [54, 55, 56–81] NRS [22, 84, 56, 70, 85–92] Personal pain history (PPH) [88] Not specified [12, 93–101] McGill Pain Questionnaire [18, 56, 70, 102, 103] POM-VAS [103] Chronic grade pain scale [56] RMDQ [87] QBPDS [104] 5 point scale [105]
Pain location	Body chart [30, 41]	Body chart [61, 67, 69, 72, 86, 106] Questionnaire [42]
Pain frequency	/	Questionnaire [68]
Pain prevalence	/	Self-report [97, 106–109] Questionnaire [57] Not specified [62]
Pain behaviour	/	Pain Behavior Scale [102]
Functional outcomes		
Function	ODI [23, 24, 36, 39, 43] DRI [23, 24, 33, 41, 43] PSFS [35, 36, 51] RMDQ [44, 51] QBPDS [27] PGQ [39, 48–50] ADL questionnaire [27, 42] Majeed score [45] Not specified [52] VAS [28]	ODI [21, 56, 66, 70, 86, 91, 108, 110] DRI [56, 106] PSFS RMDQ [19, 84, 56, 62, 70, 87, 102] QBPDS [56, 102] PGQ [56, 70, 86] VAS [18, 19, 102, 106] Likert scale [22, 63, 69, 76] Majeed score [111] BPFS [78] Not specified [90, 95, 96, 100, 112, 113] Inventory of functional status after childbirth [69] Endurance of walking/sitting/standing – self report [111]
Functional mobility	Functional load transfer tests [20] TUG test [37]	The pregnancy mobility index (PMI) [73, 82]
Physical activity levels	/	Pregnancy Physical Activity Questionnaire (PPAQ) [67, 92] Exercise diaries [110] Self-report [57]
Disability	ODI [38, 47] Not specified [52]	ODI [68, 72, 75, 77, 82, 85] RMDQ [73, 74, 89, 92] QBPDS [88] PGQ [92] DRI [57, 59, 71, 81] Not specified [12, 58, 93, 95, 98, 114] Self-report interview [109] Bournemouth disability Questionnaire (BDQ) [90]
Work disability	/	Not specified [100]
Quality of Life (QOL)/health status		
QoL	SF-36 [38, 48] EuroQol/EQ-5D [23, 24, 48, 49] EQ-VAS [23] NHP [40] Hopkins symptom checklist (HSCL) [43] Not specified [34]	SF-36 [56, 86] EuroQol/EQ-5D [56, 68, 86] EQ-VAS [68] Not specified [114] NHP [56] Assessment of QOL Questionnaire [56] WHO-QOL questionnaire [75] SF-12 [62] ODI [64]
Health status / general health	SF-36 [33, 48] EuroQol [47] SF-8	SF-36 [18, 19, 102, 103] EuroQol/EQ-5D [18, 102] SF-8 [89] Not specified [88, 96, 100]
Perceived health	NHP [29]	/
Other		
Patient satisfaction (with treatment/ life satisfaction)	Likert scale [27, 115] Patient report [38]	Questionnaire [68, 86] Not specified [56, 95, 96, 100] Satisfaction with life scale (SWLS) [63] Verbal self-rating [90]
Perceived improvement	Likert scale [29]	IPA questionnaire (Effect on autonomy/participation) [18, 19, 102] Global effect 7-point scale [18, 19, 102] Likert scale [56, 62, 81, 86] VAS [59] Questionnaire [86] Patient’s Global Impression of Change test [85, 91] Percentage improvement reported by patient [90] Not specified [58, 93, 96]
Patient expectations of treatment	/	VAS [73, 102] 11 NRS no expectation to full recovery) [86]
Psychological Outcomes		
Fear avoidance/ fear of movement	/	FABQ [57, 72, 90] Tampa Scale for Kinesiophobia [18, 19, 102] Not specified [56]
Pain catastrophizing	/	Pain Catastrophizing Scale [102] Not specified [56]
General mental health	/	Beck Depression inventory [102] Multidimensional Personality Questionnaire [102]
Anxiety	/	The State Trait Anxiety Inventory (STAI) [21, 67, 71, 73]
Wellbeing	/	VAS [68] Not specified [96]
Depression	/	Postpartum Depression Scale [69] Goldberg Depression inventory [62]
Confidence	11 point numerical rating scale [48, 49]	/
Self-efficacy	Pain self-efficacy Questionnaire [35]	/
Sleep/fatigue outcomes		
Trouble sleeping	/	Self-report (frequency of waking/ delayed onset of sleep) [85, 86]
Fatigue (morning and evening)	VAS 0–100 (encouraged to complete each week on same day at same time) [29]	/

**Table 5:** Outcomes and outcome measurements identified in the ‘Resource-use/ economic impact’ domain for PGP and LPP respectively

Resources Used/ Economic Impact	PGP studies (*n* = 33)	LPP studies (*n* = 74)
Sick leave/ temporary occupational incapacity	Not specified [23–25] Self-report [47] Diary [26] Questionnaire [26]	Not specified [54, 56–58, 93–96] Reduction in requirements [59] Self-report [12, 60, 61, 97, 106, 107] Registered by two obstetricians at each visit [85] Questionnaire [98] Likert scale [62]
Analgesia use	Not specified [115]	Over the counter and prescribed [86] Not specified [22, 63, 93, 112, 114] Diary [64] Self-report [56, 62, 85, 87]
Cost		Cost diary (physical activities, healthcare utilisation, sick days) [18] Time of work for appointments, how work was covered, time impact of treatment on other activities, child care costs, accompanied appointments, mode of transport, transport costs [86] Incremental cost per day without pain (including direct and indirect costs) [108]
Work performance		Work status, time taken off work because of LBP, performance at work [86]
Healthcare utilisation		Consultations, investigations and treatments [86] Questionnaire [87]

**Table 6:** Outcomes and outcome measurements identified in the ‘Pathophysiological manifestations’ domain for PGP and LPP respectively

Pathophysiological manifestations	PGP studies (*n* = 33)	LPP studies (*n* = 74)
Pain location/ pain provocation	Topographic representation [27] Specific tests for SIJ region/ pubic symphysis [20, 28–30] Physical exam [31]	Physical exam tests [65, 85, 86, 88, 116] Physical exam [66]
Recovery of symptoms	Physical exam [32]	
Posture	Postural analysis [20]	Tests for levels of ASIS and PSIS [116]
Continence	International Consultation on Incontinence Questionnaire Short Form (ICIQ) [48, 49]	Set of purposely devised questions [67]
Pubis symphysis mobility	Radiographic examination - Chamberlian method [29]	
Muscle function (strength/ endurance)	Isometric trunk extensor/flexor tests [33]	Pelvic floor muscles: surface electromyography [68] Hip extensors (max voluntary extension): dynamometer [68] Back extensors/flexors: isometric endurance timed tests [68] PFM strength – Vaginal balloon catheter [106]
Gait speed/endurance	6MWT [33]	Timed 20 m walk test [68]
Flexibility	/	Digital forward flexmeter (HRS-220, Japan) [69]
Anthropometric outcomes	/	Weight (KG) [57, 104] BMI (KG/m2) [57]
Pregnancy outcomes / maternal outcomes	Antenatal, intrapartum, neonatal and infant data that are normally registered in the Medical Birth Register [115]	Maternal: Gestation week at delivery, live births, length of labour, induction required, mode of delivery, episiotomy or a perineal tear, estimated blood loss at birth, antenatal and postnatal haemoglobin count, pain relief during labour. Neonatal: Gender, weight, Apgar score at 1 and 5 min, admittance to neonatal unit [86] Apgar score, birth weight, perinatal loss [84] Apgar scores [67] Delivery/labour [24, 105] Gestational week of delivery [105]
Surgical outcomes (fluoroscopy time, insertion time for guide wires, operation time, screw position)	Not specified [117] Post-op CT scan [117]

**Table 7:** Outcomes and outcome measurements identified in the ‘Adverse events’ domain for PGP and LPP respectively

Adverse Events	PGP studies (*n* = 33)	LPP studies (74)
Adverse events (not specified)	Patient Questionnaire [115]	Case reports by physio [86] Identified by trialist [12] Not specified [56, 59, 70, 71, 93, 95] Questionnaire [85, 104]
Post-op complications	Not specified [117]	
Fetal outcome		Apgar score, birth weight, perinatal loss [84]
Safety of women and children		Not specified [114]

The differences in the number of outcomes reported in studies on PGP and studies on LPP by core domain are outlined in the Table 8. Notable, psychological outcomes and economic outcomes were more commonly measured in LPP studies compared to PGP studies. A further comparison of the different outcomes reported in each domain between PGP and LPP studies is outlined in Additional file 4.

**Table 8:** Outcome count by core domain for PGP and LPP studies

Core domain *Subgroup*	PGP studies (*n* = 33): No. of outcomes (%)	LPP studies (*n* = 74): No. of outcomes (%)
Life Impact	13 (50%)	22 (58%)
*Pain-related outcomes*	*2 (8%)*	*5 (13%)*
*Functional outcomes*	*3 (12%)*	*5 (13%)*
*QoL/ health status*	*3 (12%)*	*2 (5%)*
*Other*	*2 (8%)*	*3 (8%)*
*Psychological outcomes*	*2 (8%)*	*6 (16%)*
*Sleep/ fatigue outcomes*	*1 (4%)*	*1 (3%)*
Resource-use/ economic impact	2 (8%)	5 (13%)
Pathophysiological manifestations	9 (35%)	8 (21%)
Adverse events	2 (8%)	3 (8%)

## Discussion

A large number of primary intervention studies (*n* = 76) and systematic reviews (*n* = 31) were identified. A total of 46 outcomes were measured across all studies. The majority of outcomes related to the ‘life impact’ core domain of the OMERACT framework. This would be expected considering the nature and main symptoms of PGP and LPP. Within the life impact core domain, pain intensity was the most commonly reported outcome in both PGP and LPP studies, followed by the outcomes function and disability. Fifteen (20%) studies on LPP included psychological outcomes versus only three (9%) PGP studies. This is likely because LPP includes LBP, which has had a strong psychosocial focus within the literature the past few decades, including on aspects such as fear avoidance and catastrophising. It might be that PGP is often perceived as a transient condition related to pregnancy and researchers therefore assess fewer psychosocial factors that are involved in developing chronicity. However, not all women recover and PGP can persist postpartum [2, 5-7, 118]. Moreover, PGP has been associated with psychological factors including emotional distress [119], depression [118, 120] and anxiety [118]. Only 14 studies/reviews (13%) examined any adverse events. This is contrary to current recommendations to always assess adverse events for any intervention study or systematic review [121, 122].

A range of outcome measurements were used across studies to measure certain outcomes. For example, pain intensity alone was measured using 10 different outcome measurement instruments, and function was examined using 13 different tools across the studies. This emphasises not only the need for a COS but also for consensus on how to measure the identified COS. This systematic review will contribute to a list of initial outcomes to be included in a multistakeholder, international Delphi survey to reach consensus on a PGP-COS. Subsequently, the next part of the PGP-COS study will determine ‘how’ best to measure the developed COS [13].

This systematic review also showed that the included intervention studies/reviews often use different terminology to describe the same outcomes. For example, when examining the measurement tools for the outcomes ‘function’ and ‘disability’, the same tools are frequently used. While some studies use the term ‘function’ and others ‘disability’, most studies do not provide a clear definition of the terms. Another example of where there is clearly inconsistency in terminology and a lack of definitions in original manuscripts is for the outcomes ‘quality of life’ and ‘health status’. Again, the same measurement instruments tend to be used and terms seem to be used interchangeably across different studies. This observed inconsistency strengthens the rationale for the development of an agreed PGP-COS.

Chiarotto et al. [123] published a COS for non-specific LBP in 2015 and, while there was some overlap in findings, the list of outcomes they identified from the LBP literature differed significantly from our findings of the outcomes measured in the PGP/LPP literature. They identified the following outcomes in LBP studies that were not identified in our review of PGP/LPP studies: death, cognitive functioning, social functioning, sexual functioning, satisfaction with social role and activities, pain quality, independence (Life impact); informal care, societal services, legal services (Resource-use/ economic impact); muscle tone, proprioception, spinal control, and physical endurance (Pathophysiological manifestations). Outcomes that we identified in this review of PGP/LPP studies but that were not identified in the review of the outcomes measured in the LBP literature [123] were: Self-efficacy, confidence, patient expectations of treatment (Life impact); and anthropometric measures (weight/height), pregnancy and maternal outcomes, surgical outcomes (Physiological manifestations). Some of the observed differences could be put down to differences between PGP and LBP. However, differences in outcomes seem largely arbitrary instead of relating to the distinguishing features of PGP and LBP. Similarly, when comparing studies examining PGP only with studies examining LPP in this systematic review, the reason for the observed discrepancies in the outcomes chosen by studies’ authors are mostly unclear. This supports using the outcomes identified in this review only as an initial list for the consensus process to develop a PGP COS, allowing for other outcomes to be added by all stakeholders including patients, clinicians, researchers, service providers and policy makers.

## Conclusions

Studies and systematic reviews examining the effectiveness of interventions for PGP and LPP assess a range of outcomes, predominantly pain intensity and disability/function, and use a large variety of outcome measurement instruments. Few studies examine adverse events and economic outcomes. Not only do different studies often measure different outcomes, authors also rarely define outcomes and terminology for outcomes varies, making comparison of study findings very difficult.

## Supplementary information

**Additional file 1.** Search strategy. A detailed outline of the search strategy of this systematic review including the databases searched and exact search terms used.

**Additional file 2.** Charateristics of included studies. A detailed description of the studies/systematic reviews that were included in this systematic review.

**Additional file 3.** Quality of reporting. The results of the assessment of the quality of reporting in the individual studies included in this systematic review.

**Additional file 4.** Comparison of outcomes identified in PGP and LPP studies for each core domain. The outcomes that were identified in studies examining PGP only are compared to the outcomes identified in studies including patients with LPP. This comparison has been presented by core domain.

## Data Availability

Not applicable.

## Abbreviations

6MWT: 6 min walk test
ADL: Activities of daily living
ASIS: Anterior superior iliac spine
BDQ: Bournemouth disability questionnaire
BMI: Body mass index
BPFS: Brief psychiatric rating scale
COS: Core outcome set
CT scan: Computerised tomography scan
DRI: Disability rating index
EQ-VAS: EurQoL visual analogue scales
EurQoL – EQ-5: EurQoL 5 dimensions
HSCL: Hopkins symptom checklist
ICIQ: International consultation on incontinence questionnaire
KG: Kilogram
LBP: Low back pain
LPP: Lumbopelvic pain
NHP: Nottingham health profile
NRS: Numeric rating scale
ODI: Oswestry disability index
OMERACT: Outcome measures in rheumatology
PFM: Pelvic floor muscles
PGP: Pelvic girdle pain
PGQ: Pelvic girle questionnaire
PMI: Pregnancy mobility index
POM-VAS: Pain-O-meter visual analogue scale
PPAQ: Pregnancy Physical Activity Questionnaire
PRISMA: Preferred reporting items for systematic reviews and meta-analyses
PSFS: Patient-specific functional scale
PSIS: Posterior Superior Iliac Spine
QBPDS: Quebec back pain disability scale
QoL: Quality of life
RCT: Randomised controlled trial
RMDQ: Roland Morris disability questionnaire
SF-12: Short form 12
SF-36: Short form 36
SIJ: Sacroiliac joint
SWLS: Satisfaction with life scale
TUG: Timed up and go test
VAS: Visual analogue scale
