# Supplementary material for: Outcomes and outcomes measurements used in intervention studies of pelvic girdle pain and lumbopelvic pain: a systematic review
Source: Chiropr Man Therap. 2019 Nov 5;27:62. doi: 10.1186/s12998-019-0279-2 (PMC6829811; doi:10.1186/s12998-019-0279-2)
Supplement: Supplementary file 1 — Additional file 1. Search strategy. A detailed outline of the search strategy of this systematic review including the databases searched and exact search terms used. [file 12998_2019_279_MOESM1_ESM.docx]

| **Additional file 1: Search Strategy** | | | |
| --- | --- | --- | --- |
| **Database (Date)** | **Filters** | **Search** | **Number of citations** |
| PubMed  (11 May 2018) | None | (“low back pain”[Mesh] OR “pelvic pain”[Mesh] OR “sacroiliac joint”[Mesh] OR “pelvis”[Mesh] OR “pubic symphysis”[Mesh] OR “sacrum”[Mesh] OR pelvic OR pelvis OR sacroiliac OR “sacro iliac” OR sacral OR sacrum OR “pubic symphysis” OR “symphysis pubis” OR symphyseal OR lumbopelvic OR lumbar OR “low back”) AND (“pain”[Mesh] OR pain OR instability OR insufficiency OR subluxation) AND (“pregnancy”[Mesh] OR “pregnancy complications”[Mesh] OR “postpartum period”[Mesh] OR “parturition”[Mesh] OR pregnancy OR “ante natal*” OR prenatal* OR antenatal* OR “pre natal*” OR “prenatal*” OR birth* OR childbirth OR perinatal* OR “peri natal*” OR postpartum OR “post partum” OR postnatal* OR “post natal*”) | 5176 |
| EMBASE  (11 May 2018) | (without MEDLINE) | (('low back' OR 'pelvic girdle' OR 'sacroiliac joint' OR 'sacroiliac joints' OR pelvis OR pelvic OR lumbar OR pelvic OR sacroiliac OR 'sacro iliac' OR sacral OR sacrum OR coccyx OR coccygeal OR 'symphysis pubis' OR 'pubic symphysis' OR symphyseal OR lumbopelvic OR back) AND (pain OR instability OR insufficiency OR subluxation) AND (pregnancy OR parturition OR 'ante natal' OR 'ante natally' OR prenatal* OR ‘pre natal’ OR ‘pre natally’ OR antenatal* OR birth OR childbirth OR perinatal* OR ‘peri natal’ OR ‘peri natally’ OR postpartum OR 'post partum' OR postnatal* OR ‘post natal’ OR ‘post natally’) NOT (fibroid* OR endometriosis OR cyst* OR haemorrhage OR neoplasm OR cancer OR malignant OR 'pelvic inflammatory disease' OR salpingitis OR osteoporosis OR placenta OR placental OR ultrasound)) | 2619 |
| Cochrane Library  (11 May 2018) | Cochrane reviews, other review, trials | (pelvic OR pelvis OR sacroiliac OR “sacro iliac” OR sacral OR sacrum OR “pubic symphysis” OR “symphysis pubis” OR symphyseal OR lumbopelvic OR lumbar OR “low back”) AND (pain OR instability OR insufficiency OR subluxation) AND (pregnancy OR “ante natal*” OR prenatal* OR antenatal* OR “pre natal*” OR “prenatal*” OR birth* OR childbirth OR perinatal* OR “peri natal*” OR postpartum OR “post partum” OR postnatal* OR “post natal*”) | 565 |
| PeDRO  (11 May 2018) | None | Abstract/title: pelvi*  Body part: lumbar spine, sacro-iliac joint or pelvis  Subdiscipline: musculoskeletal  Methods: Systematic review, clinical trial | 137 |
