# Supplementary material for: Outcomes and outcomes measurements used in intervention studies of pelvic girdle pain and lumbopelvic pain: a systematic review
Source: Chiropr Man Therap. 2019 Nov 5;27:62. doi: 10.1186/s12998-019-0279-2 (PMC6829811; doi:10.1186/s12998-019-0279-2)
Supplement: Supplementary file 2 — Additional file 2. Charateristics of included studies. A detailed description of the studies/systematic reviews that were included in this systematic review. [file 12998_2019_279_MOESM2_ESM.docx]

**Additional file 2: Characteristics of included studies**

1. *Characteristics of included studies on PGP****:***

| **Author (year)** | **Country** | **Study Design** | **Participants (n)** | **PGP definition** | **Intervention(s)** | **Control(s)** |
| --- | --- | --- | --- | --- | --- | --- |
| Almousa et al (2018) | UK | Systematic Review | Women who suffered from PGP during pregnancy or post-partum period or both. | PGP related to pregnancy, musculoskeletal cause, pain between posterior iliac crest and gluteal fold. | Stabilising exercises | Any control group |
| Almousa et al (2015) (conference abstract) | Greece | Systematic Review | Women who suffered from PGP during pregnancy or post-partum period or both. | Not defined. | Stabilising exercises | Any control group |
| Barfoot et al (2015) (Conference abstract) | UK | Pilot RCT | Women between 14 and 32 weeks gestation (n=61) | PGP as diagnosed on physical exam using validated tests. | (1) once off individual session, advice and exercise  (2) 6 week group course   \| (3) 6 week individual course \| \| --- \| | Once off group session advice and exercise (usual care) |
| Bertuit et al (2018) | Belgium | RCT | Pregnant women 25-35 years, from 18th week pregnancy, (n= 46) | Pain in the SIJs and/or pubic region, verified by a positive result for at least half of the following set of tests (P4 test, Patrick Faber’s test, Trendelenburg modified test, pain provocation tests and active straight leg raise test) | Pelvic belt type 1 (Ortel-P Thuasne) or 2 (LombaMum Thuasne) | No belt |
| Bhandiwad et al (2015) (Conference abstract) | India | Non-controlled clinical trial | Women diagnosed with PGP in second or third trimester of pregnancy. (n=12) | Not specified | Pregnancy-specific exercise program 3 day per week for 8 weeks | No control |
| Bromley and Bagley (2014) | UK | Systematic review | Women with a diagnosis of pregnancy-related PGP (including pregnancy-related pain in the lumbosacral region, sacroiliac joints and symphysis pubis) | Pregnancy-related pain in the lumbosacral region, sacroiliac joints and symphysis pubis | Physiotherapist-guided exercise, including: core stability exercises, pelvic floor muscle exercises, hydrotherapy, general exercise | Not specified |
| Cameron et al (2017) (Conference abstract) | England | Single case experimental design | Post-partum women with chronic PGP (n = 8) | PGP as diagnosed by pain referral map and several pain provocation tests. | Combined physiotherapy and a dynamic elastomeric fabric orthosis. | Standard care |
| Cameron et al (2015) (Conference abstract) | UK | RCT | Pregnant women with PGP | Diagnosed using pain referral map and a battery of pain provocation tests, in line with the European Guidelines | Rigid pelvic belt (plus standardised advice) | Customised Dynamic Elastomeric Fabric Orthoses (DEFO) (plus standardised advice) |
| Clarkson et al (2016) (Conference abstract) | UK | Two-armed randomised control feasibility study | Women with Pregnancy-related PGP (n=40) | Not defined | Standard care + penetrating Korean hand acupuncture (6 sessions) | Standard care + non-penetrating Korean hand acupuncture (6 sessions) |
| Depledge et al (2005) | New Zealand | Randomised masked prospective clinical trial | Pregnant women with pain in symphysis pubis with/without radiation to groin, referred for physio (n=90) | Pain in symphysis pubis with/without radiation to groin, insidious onset during pregnancy, positive ASLR. | (1) Exercise + rigid support belt  (2) Exercise + non-rigid support belt | Exercise |
| Elden et al (2008)(a) | Sweden | Randomised double blind control study | Healthy pregnant women with singleton foetuses, between 12 and 29 weeks gestation, with a clinical diagnosis of PGP who scored ≥50 on a 100-mm visual analogue scale (VAS) (n=115) | PGP between posterior iliac crest and gluteal fold, physical exam including ASLR test and pain provocation tests, score ≥50 on a 100-mm visual analogue scale (VAS) during baseline week. | Standard care plus penetrating acupuncture 12 treatments | Standard care plus non-penetrating acupuncture 12 treatments |
| Elden et al (2008)(b) | Sweden | Follow-up study for RCT (Elden et al 2005) | Healthy women at 12 - 31 completed gestational weeks, with singleton foetuses and defined PGP. | PGP defined as: weight-bearing-related pain in the posterior pelvis, deep in one or both gluteal areas. Pain-drawing with markings in the gluteal area distal and lateral to L5-S1 with /without radiating pain on the posterior thigh but not down to the foot. Pain-free intervals with sudden pain attacks. Pain when turning in bed. A free range of motion in the hips and spine. No nerve root syndrome. A positive P4-test. | (1) Standard treatment plus acupuncture  (2) Standard treatment plus specific stabilising exercises | Standard treatment (6 weeks) |
| Elden et al (2005) | Sweden | Randomised single blind trial | Healthy women at 12-31 completed gestational weeks, with singleton foetuses and defined pregnancy-related PGP. | PGP as diagnosed by: P4 test, Patrick’s faber test, a modified Trendelenburg’s test, Lasegue test, and palpation of the symphysis pubis | (1) Standard treatment plus acupuncture  (2) Standard treatment plus specific stabilising exercises | Standard treatment (6 weeks) |
| Elden et al (2008)(c) | Sweden | RCT | Healthy women at 12–31 completed gestational weeks with singleton foetuses and diagnosed with PGP. | PGP diagnosed according to Ostgaard’s criteria: time- and weight bearing related pain, pain-free intervals with sudden pain attacks, pain when turning in bed, a free range of motion in the hips and spine, no nerve root syndrome and a positive posterior pelvic pain provocation test | (1) Acupuncture + standard care  (2) Stabilising exercises + massage + standard care | Standard care |
| Elden et al (2013) | Sweden | Single blind multicentre RCT | Healthy pregnant women with singleton foetuses at 12–29 completed gestational weeks experiencing moderate evening pain, i.e.  equal to or exceeding 40 mm on a 100-mm pain VAS during the baseline week. | PGP: pain between posterior iliac crests and the gluteal folds, particularly in the vicinity of the sacroiliac joints along with or only in the symphysis pubis, positive pain drawing with markings in the symphysis and/or in the gluteal areas distal and lateral to L5–S1, with/without radiating pain on to the posterior thigh but not to the foot, diminished endurance capacity for standing, walking and sitting, free range of motion in the hips and spine, and no nerve root syndrome, i.e. exclusion of lumbar causes such as a positive posterior pain provocation test or the symphysis pubic pressure test | Standard treatment plus craniosacral therapy. | Standard treatment (advice, information, home training program, elastic belt.) |
| Flack et al (2015) | New Zealand | Unblinded, single centre, 2-arm randomised group study | Women were eligible to take part in the study if they were pregnant, at least 18 years of age, had experienced pubic symphysis pain for at least two weeks. | Pubic symphysis pain which was worse than any concurrent posterior pelvic pain, and had a positive response to at least two of three clinical tests: reproduction of pain from palpation, modified Trendelenburg’s test, active straight leg raise test | Rigid support belt (3 weeks) | Flexible support belt (3 weeks) |
| Gausel et al (2017) | Norway | RCT | Pregnant women from 18 weeks to 29 weeks at recruitment, with dominant one sided PGP (n=56) | Those reporting PGP at baseline verified by pain drawings were invited for a clinical exam to confirm diagnosis. If pain present and lumbar pain excluded, and one of following test positive: P4 test, Patrick’s Faber test, palpation of the long dorsal sacroiliac ligament, Gaenslen’s test, palpation of the symphysis, modified Trendelenburg test of the pelvic girdle and ASLR test. | Chiropractor treatment: manipulation, mobilization, soft tissue treatment, exercises, and advices chosen by the chiropractor to fit each participant individually. | Conventional primary care, no restrictions. |
| Gupta et al (2015) (Conference abstract) | Australia | Randomised cross-over blinded sham-controlled trial | (n=22) pregnant women with PPGP | PPGP and no other significant or serious pathology or obstetric related complication. (no definition provided) | Therapist-assisted exercise (EX) +TENS, or TENS + EX (opposite order) followed by standard physiotherapy (education, manual therapy, exercise, pelvic belt application) | No control |
| Haugland et al (2006) | Norway | RCT | Pregnant women with posterior pelvic pain and/or pain from the symphysis pubis between the 18th and 32nd week of gestation (n=569) | PGP: Positive P4 test and/or positive symphysis pressure test. Aggravated by minimum two of: walking>10mins, standing>10mins, climbing two flights stairs, turning in bed. Negative neuro back exam. | Education program in small groups for 4 weeks | No treatment |
| Kibsgard et al (2014) | Norway | Single-subject research design study with repeated measurements | Patient with PGP, unresponsive to physiotherapy over time (n=9) | PGP diagnosis criteria: pain in one or more pelvic joints, positive in min 2 out of 5 clinical tests (P4, ASLR, palpation of long dorsal SI-ligament, modified Trendelenburg, palpation of symphysis). ODI > 40 and/or VAS> 50 | Surgical procedure: unilateral anterior SIJ fusion and fusion of symphysis pubis | No control group |
| Kuciel et al (2017) | Poland | Preliminary study - non-controlled clinical trial | Pregnant women, age: 20–35 years old; 18th–34th week of pregnancy, with diagnosed PGP (n=24) | At least one positive test out of three applied in the diagnoses of PPGP; long dorsal ligament test, P4 test, Trendelenburg test. Negative Straight Leg Rise test. | Kinesiotaping | No control group |
| Ladfors et al (2004) | Sweden | RCT | (n=386) pregnant women with confirmed isolated PGP | PGP confirmed by physical examination by an independent observer | (1) Standard treatment + acupuncture  (2) Standard treatment + stabilising exercises | Standard care |
| Lund et al (2006) | Sweden | RCT | Pregnant women with pelvic girdle pain, median gestational age 26 weeks | PGP for at least 2 weeks. Pain intensity last week, rated on VAS 0-100: ≥60 Increased pelvic pain by walking, turning from one side to the other in bed, or rising from sitting to standing. Physical examination confirming provoked pelvic pain: 1) in one of three tests: 4P test, standing on one leg, Patrick’s Faber test 2) in palpating tissue over / the sacroiliac joints, the symphysis pubis, or mm. gluteus maximus/medius | (1) Superficial acupuncture x 10 treatments  (2) Deep acupuncture x 10 treatments | No control |
| Melkersson et al (2017) | Sweden | Randomised single blind pilot trial | Women in weeks 12 to 31 of pregnancy who had PPGP as determined by specific provocation tests and women with only LBP were excluded. | PGP tests: the posterior pelvic pain provocation test, Patrick (FABER) test, ASLR test, modified Trendelenburg test, and palpation of the symphysis pubis. The precise area of pain was indicated on an anatomic pain drawing, | Foot manipulation treatment + information on PPGP +home exercises | Comparative treatment including massage technique +information +home exercises |
| Mens et al (2000) | The Netherlands | RCT | Post-partum women with most recent pregnancy between 6 months and 6 weeks of beginning of study. | PGP: pain experienced between the plane through the 4 superior iliac spines and the horizontal plane through the inferior border of the pubic symphysis. Influenced by position and locomotion. Localised posteriorly and anteriorly, onset during pregnancy or within 3 weeks of delivery. | Information video re PGP prognosis and ergonomic advice + exercises to increase the force of the diagonal trunk muscle systems | (1) Information video re PGP prognosis and ergonomic advice + training of the longitudinal trunk muscle systems (rectus abdominis muscle, longitudinal parts of the erector spinae muscle, and quadratus lumborum muscle)  (2) Information video re PGP prognosis and ergonomic advice + told to refrain from exercise |
| Nilsson-Wikmar et al (2005) | Sweden | RCT | Pregnant women up to gestation week 35 with defined PGP (n=118) | Pain provocation tests for the pelvic joints were used: palpation over the sacroiliac joints, iliac gapping/distraction test, iliac compression/ approximation test, Patrick test, posterior pelvic pain provocation test, symphysis pubis pressure test and sacral apex pressure test. Women who tested positive in at least 3 pelvic pain provocation tests including the symphysis, while testing negative for pain in the lumbar spine area including radiating pain, were included. | (1) A home exercise program consisting of 3 exercises aiming to stabilize the muscles around the pelvic girdle + same information as control.  (2) In -clinic training program comprised of 4 different strengthening and stabilization exercises with different pieces of equipment; the lateral pulls, standing leg-press, sit-down rowing, and curl-ups. Twice a week until gestation week 39 + information as in control. | Information about the condition including anatomy, body posture, and ergonomic advice and were provided with a non-elastic sacroiliac belt |
| Ribnikar et al (2015) (Conference abstract) | Slovenia | RCT | Women in week 18- 37 of pregnancy, with confirmed PGP. (n=40) | PGP confirmed as diagnosed by at least 4/6 positive clinical tests in line with European guidelines. | Physiotherapy advice + non-rigid belt | Physiotherapy advice alone |
| Schep et al (2004) | The Netherlands | prospective controlled study | 24 patients with postpartum pelvic pain syndrome | Not defined | Fluoroscopy-based computer-assisted surgery for insertion of Cannulated Iliosacral Screws | Conventionally operated for insertion of cannulated iliosacral screws |
| Stuge et al (2004) + Stuge et al (2004) 2 year follow up. | Norway | RCT | Women with postpartum PGP (6-16 weeks post-delivery) (n=81) | PGP criteria: PGP located distal and/or lateral to the L5–S1 area in the buttocks and/or in the symphysis; positive P4 test; and/or ASLR test; pain provocation of long dorsal sacroiliac ligament; and pain provocation of the symphysis by palpation and by modified Trendelenburg test. | Physiotherapy with specific stabilising exercises | Physiotherapy without specific stabilising exercises |
| Torstensson et al (2013) | Sweden | RCT | Women with persistent pregnancy related PGP with onset during pregnancy and of 6 months to 7 years duration after delivery. (n=36) | PGP criteria: ongoing pain in the sacral region reported on a pain drawing, reported pain intensity at present or worst during the past week between 30 and 70 mm on a 100-mm horizontal VAS; at least one out of three positive pain provocation tests; and pain elicited on internal palpation at the ischiadic spine, at least unilaterally. | Injection with slow-release triamcinolone and lidocaine | Injection with saline and lidocaine |
| Torstensson et al (2009) | Sweden | RCT | Postpartum women with pregnancy-related sacral low back pain. (n=36) | Ongoing pain in the sacral region with onset during pregnancy and 6 months to 7 years duration after delivery, reported pain intensity at present between 30 and 50 mm on a 100-mm horizontal VAS, at least 1 positive pain provocation test out of 3, and pain elicited on internal palpation at the ischial spine at least unilaterally. | Injection treatment of either a compound of 1 mL triamcinolone, 20 mg/mL, and 1 mL lidocaine hydrochloride 10 mg/mL into sacrospinous ligament insertion on ischial tuberosity. | Sham injection treatment; 0.99 mL saline solution 9 mg/mL, 1 mL lidocaine hydrochloride, and 0.01 mL fat emulsion into sacrospinous ligament insertion on ischial tuberosity. |
| Vaidya (2018) | India | RCT | Pregnant participants age 20- 35 with uncomplicated pregnancy and posterior pelvic pain unilateral and bilateral (n=30) | Posterior pelvic pain: positive FABERS test and score on VAS >50mm | SIJ mobilisation based on Maitland concept (5 sessions) | TENS (5 sessions) |
| Weil et al (2008) | USA | Retrospective study | Women treated for postpartum pelvic pain (n=19) | Anterior pain in the area of the symphysis pubis or groin starting immediately during delivery (n=17) anterior pain during the second trimester of pregnancy and was clinically diagnosed with symphysis diastasis (n=1) + posterior pelvic pain in the buttock or SI joint (n=11) | (1) Non-operative treatment: physiotherapy, which involved gait training and pelvic floor–strengthening exercises  (2) Surgical intervention | No control |

1. *Characteristics of included studies LPP:*

| **Author (year)** | **Country** | **Study Design** | **Participants (n)** | **PGP/LPP definition** | **Intervention(s)** | **Control(s)** |
| --- | --- | --- | --- | --- | --- | --- |
| Abu et al (2017) | Malaysia | RCT | Low-risk pregnant women, 17 - 28 weeks amenorrhea, age 18-35. n= 145. | LBP and PGP (deep pain distal and lateral to L5/S1 over SIJ and posterior iliac crest), differentiated with pain drawing. | verbal information, back care pamphlet and paracetamol + physio exercise session (2 hours), encouraged to continue exercises at home with exercise diary for exercise and medication use for 6 weeks | Verbal information, back care pamphlet and paracetamol with diary for medication use for 6 weeks |
| Al-Sayegh et al (2010) | USA | prospective cohort | Postpartum patients reporting pain in the lower back and/or buttocks, age 18 – 45, were within 1 year of giving birth | Pain in the areas of the lower back, pelvis, buttock, and legs who were referred for physical therapy, with ODQ score had to be at least 30% | High velocity thrust technique | None |
| Balasundaram et al. (2017) | Norway | Systematic review | Post-partum women, with onset of PGP during pregnancy. (n= 341) | LBP and/or PGP related to pregnancy | Physiotherapy interventions | Any control groups |
| Barkatsa et al (2010) (Conference abstract) | Greece | Systematic review | Pregnant women with PGP or LBP | Not defined | Any interventions | Not specified |
| Bastiaenen et al (2006) | The Netherlands | RCT | Women >18 years, PGP and/or LBP onset during pregnancy or within 2 weeks of delivery. (n=126) | PGP (including low back pain), restricted in ADLs, delay in recuperation post pregnancy due to pain. Physical exam to exclude pathologies. | Individualised self-management 7-9 sessions, 30 min. sessions (starting approx. 3 weeks postpartum) | Usual care |
| Bastiaenen et al (2008) (Long term follow up of Bastiaenen et al (2006)) | The Netherlands | RCT | Women >18 years, PGP and/or LBP onset during pregnancy or within 2 weeks of delivery. (n=126) | PGP (including low back pain), restricted in ADLs, delay in recuperation post pregnancy due to pain. Physical exam to exclude pathologies. | Individualised self-management 7-9 30 min. sessions (starting approx. 3 weeks postpartum) | Usual care |
| Bastiaenen et al (2004) *protocol for 2006 paper | The Netherlands | RCT | Women >18 years, PGP and/or LBP onset during pregnancy or within 2 weeks of delivery. | PGP (including low back pain), restricted in ADLs, delay in recuperation post pregnancy due to pain. Physical exam to exclude pathologies. | Individualised self-management 7-9 30 min. sessions (starting approx. 3 weeks postpartum) | Usual care |
| Bennett (2014) | UK | Systematic review | Women up to one year post-partum with LBP or PGP | LBP and/or PGP | Any form of exercise intervention except PFME's in isolation | Not specified |
| Bishop et al (2016) + Foster et al (2016) | UK | pilot RCT | Women with pregnancy-related LBP defined as self-reported pain in the lumbar area (between the 12th rib and the gluteal fold) with or without PGP, >18 years old, between 13 to 31 weeks gestation. (n=125) | Pregnancy-related LBP with or without PGP. Women with PGP only were excluded. | (1) True acupuncture + standard care  (2) Standard care + non-penetrating acupuncture | Standard care |
| Butel et al (2016) (Conference abstract) | France | RCT | Pregnant women with PGP or LBP (n=200) | Not specified | Acupuncture 5 sessions + standard care | Standard care |
| Close et al 2014 | UK | systematic review of RCTs | Pregnant women with low back or pelvic pain | Not specified | Complementary and alternative medicines | Physiotherapy, sham, stabilising exercises, exercise, usual care. |
| Close et al (2016) | UK | RCT | First time pregnant women, ≥18years of age, LBP and/or PGP, 26-29 weeks gestation (n=90) | Not specified | Reflexology (30 mins/week for 6 weeks) | Usual care |
| Daly et al (1991) | USA | intervention study | Pregnant women with low back pain due to sacroiliac subluxation. (n=11) | Diagnostic criteria: sacral pain, positive Piedallu's sign, positive pelvic compression, asymmetry of ASIS. Tests to confirm: SLR, flexion block in supine, positive Patricks test, pain at Baer's point. | Manipulation technique. | No control |
| Ee et al. (2008) | Australia | Systematic review | Pregnant women with pelvic or back pain | Not defined | Acupuncture | Physiotherapy, physical exercise, no treatment, standard treatment |
| Eggen et al (2012) | Norway | Observer-blinded RCT | Pregnant women age 18 -40, before gestation week 20. (n=257) | Self-report of PGP or LBP | Supervised, group-based exercise program, each session 60 minutes, and the groups trained for 16 to 20 weeks (between gestation weeks 16 and 36). | Standard care (regular visits to health centre every 4th week) |
| Ekdahl and Peterson (2010) | Sweden | Intervention study | Healthy pregnant women with pelvic or low back pain, (n=40) | PGP or low back pain as diagnosed at maternity healthcare setting after clinical history taking | (1) Acupuncture commenced at week 20 (8 treatments)  (2) Acupuncture commenced at week 26 (8 treatments) | No control |
| Ferreira and Alburquerque-Sendin (2013) | Brazil | Systematic review | Women with PGP or LBP during pregnancy or pregnancy-related after delivery. (n=341) | PGP and/or LBP | Various physiotherapy interventions (self-management, education, stabilisation exercises) |  |
| Fisseha and Mishra (2016) | Ethiopia | Systematic review and meta-analysis | Pregnant women >18 years | Self-report of lumbopelvic pain | Group training or group exercise | Usual care and individual training |
| Franke et al (2017) | Germany | Systematic review and meta-analysis | Pregnant or postpartum adults (> 18 years and with postpartum defined in these studies from 3 to 24 months following delivery) with nonspecific LBP and/or PPP | LBP (i.e., pain between the lumbo-pelvic region and the 12th rib) and/or PPP (pain in the symphysis pubis and/or pain in the regions of one or both sacroiliac joints and pain in the gluteal region) - self report | Osteopathic manipulative therapy | No treatment, sham ultrasound, usual care. |
| George et al (2013) | USA | Prospective randomised trial | Patients 15-45 years old with a single foetus from 24-28 weeks’ gestation were evaluated by their obstetric provider for LBP, PP or both. (n=137) | The physical assessments to identify the origin of pain included the straight leg raise (SLR), posterior PP provocation test, active SLR, and long dorsal ligament test. | Multimodal approach of manual therapy, exercise, and education for LBP/PP | Standard obstetric care |
| Granath et al (2006) | Sweden | RCT | (n=390) Healthy pregnant women with either PGP or PLBP | Criteria for PGP: time- and weight bearing – related pain in the posterior pelvis, deep within the gluteal area. Positive P4 test, pain-free intervals, pain turning in bed, onset during pregnancy, positive pain drawing with markings in buttocks distal and lateral to the L5-S1 area +/- radiation to thigh. No nerve root syndrome. Criteria for PLBP: negative P4 test, pain normally prior to pregnancy, TOP back muscles, reduced/ painful motion lumbar spine | Weekly water based aerobic classes | Weekly land based aerobic classes |
| Gross et al (2012) (Conference abstract) | USA | RCT | Women with LBP or PGP during pregnancy between 24-28 weeks gestation at enrolment (n=169) | LBP and/or PGP non differentiated | Multi-modal musculoskeletal and obstetric management (MOM) | Standard obstetric care (STOB) |
| Guerreiro da Silva et al (2004) | Brazil | Prospective quasi-randomised control study | Pregnant women age 15 - 39, 15 to 30 weeks of pregnancy and at least minimal low back or pelvic pain. | Low back and pelvic pain not distinguished, self-report | Conventional treatment (paracetamol and hyoscine) + acupuncture | Conventional treatment only |
| Gutke et al 2010 (+Gutke 2011 abstract) | Sweden | RCT | Women with PGP or PGP with lumbar pain persistent 2-3 months post-partum (n=88) | Mechanical assessment of lumbar spine according to the Mechanical Diagnosis and Therapy protocol (MDT) and pelvic pain provocation tests performed in the mentioned order; distraction test, P4 test, Gaenslen’s test, compression test, sacral thrust. ASLR test, hip rotation range-of-motion test, and a neurological examination. Diagnosis PGP: ≥ 2 positive pelvic pain provocation tests, pain onset during a pregnancy or within 3 weeks from delivery and pain located distal and/or lateral to the L5–S1 area in the buttocks. | Specific stabilising exercises, 10 reps at least twice a day, progression of level of exercise throughout treatment, and meetings with physiotherapist every second week | One single telephone contact with physiotherapist to explain nature of PGP and o advise on resuming normal activities |
| Gutke et al (2015) | Sweden | Systematic review | Women with pregnancy-related lumbopelvic pain (either PGP or LBP or both) | PGP: pain between posterior iliac crests and gluteal folds and may radiate down leg, with/without pubic symphysis pain. LBP: pain below ribs and above gluteal folds, with/without radiation to legs. | Various physiotherapy interventions (acupuncture, pelvic girdle belt, group/individual exercises, water gymnastics, manual therapy, electrotherapy, yoga, education) |  |
| Hall et al (2016) | Australia | systematic review | Pregnant women of any age and at any time during the antenatal period. | Pregnancy-related low back or pelvic pain | Massage, chiropractic, and osteopathic treatments. | Usual care or standard prenatal care, progressive muscle relaxation, sham ultrasound, exercise and chiropractic neuro-emotional techniques |
| Haakstad and Kari (2015) | Norway | RCT | Sedentary, nulliparous pregnant women, mean age 30.7 years, within first 24 weeks of pregnancy (n=105) | Self-report of symptoms | Group fitness class for pregnant women twice a week, in addition to 30 min of moderate self-imposed physical activity, for a min of 12 weeks. | Usual care, neither encouraged nor discouraged for exercise |
| Hilde et al (2016) | Norway | Systematic review | Post-partum women with either PGP or combination of PGP and LBP | Onset of pain could be during pregnancy or after delivery, and based on self-report or clinical assessment. Studies with participants with purely LBP, or specific pathologies are excluded | Physiotherapy interventions | No treatment |
| Ho et al (2009) | Hong Kong | Systematic review | Pregnant women and postpartum women with PPGP or PLBP | PLBP and PPGP | Maternity support belts |  |
| Kalus et al (2008) | Australia | RCT | Women between 20 and 36 weeks of pregnancy presenting at the antenatal clinics of The Royal Women’s Hospital with lumbar back pain or posterior pelvic pain (n=94) | Diagnosis and classification of the pain was based on an oral history and on the patient’s localisation of their pain on a visual back chart. | Belly-bra garment | Tubigrip |
| Kaplan et al (2016) | Turkey | RCT | Pregnant women between age 18 and 40 years, any parity, gestational age between 10 and 30 weeks, low back pain (n=65) | Low back pain experienced anywhere from T12 to the gluteal fold without leg pain, and at least moderate pain intensity (scoring ≥4 on VAS) - self report | Kinesiotaping + paracetamol for 5 days | Paracetamol for 5 days |
| Kinser et al (2017) | USA | Systematic review | Women who presented with PR-LBP, pelvic girdle pain, or related postpartum pain | Pain occurring for more than 1 week during pregnancy and possibly continuing into the postpartum period; encompasses low back pain (LBP) and pelvic girdle pain; onset typically after 20 weeks gestation. | Yoga-based interventions, physical activity based interventions, multimodal interventions, stabilising exercises | Standard obstetric care |
| Kluge et al (2011) | South Africa | RCT | Maternal age between 20 and 40 years, any parity, gestational age between 16 and 24 weeks, low back pain/PGP experienced that started during the current pregnancy, and any degree of pain. | Low back pain experienced anywhere from T12 to the gluteal fold—with or without radiation to the knee. P4 test, palpation of sacroiliac joints and erector spinae muscles. Body diagram - differentiated PGP, LBP or both | Specific exercise program (every 2 weeks for 10 weeks plus home exercises with exercise diary)+ information/pamphlet | Information and pamphlet given |
| Kordi et al (2013) | Iran | RCT | Healthy pregnant women with pain in lumbar region radiating between gluteal fold and posterior iliac crest. Gestational age: 20–32 weeks. Mono foetus pregnancy. Age < 40 years. (n= | Diagnosis of Pelvic Girdle Pain was performed on base of pain drawing and positive result of one of two following tests for each type of patients: ∗ PATRIC’s test and posterior pelvic pain provocation test for patients with more pain around the sacro-iliac joint ∗ modifying Trendelenburg test and direct palpation of symphysis pubis test for patients with more complaints in symphysis pubis | (1) Lumbopelvic belt +information/advice  (2) Home exercise program + information/advice | Information/advice |
| Kvorning et al (2004) | Sweden | RCT | Pregnant women in third trimester of pregnancy presenting at the maternity wards with girdle or low-back pain (including mixed pain patterns) (n=72) | Not specified | Acupuncture | Untreated |
| Liddle and Pennick (2015) | Ireland | Systematic review | Pregnant women at any stage of their pregnancy with LBP, PGP or both | LBP, PGP or both | Various treatments: exercise (land- or water-based), pelvic belts, osteopathic manipulative therapy (OMT), spinal manipulative therapy (SMT), neuro emotional technique (NET), craniosacral therapy (CST), transcutaneous electrical nerve stimulation (TENS), kinesiotaping (KT), yoga, acupuncture, acupuncture plus exercises, and a multi-modal approach incorporating manual therapy, exercise and education | Not specified |
| Lillios et al (2012) | UK/USA | Systematic review | Women with complaint of LBP or PGP | PGP: pain of musculoskeletal origin between the levels of the posterior iliac crests and gluteal folds involving the anterior and/or posterior aspects of the pelvis that may radiate into the posterior thigh and may or may not be concurrent with low back pain | Physical therapy interventions that include group or individual exercise interventions | Comparison intervention or control |
| Majchrzycki et al (2015) | Poland | Systematic review | Pregnant women with back and pelvic pain | Not defined | Osteomanipulative therapy | Not control |
| Martins and Silva (2014) | Portugal | RCT | Pregnant women (age range, 14–40 years) who reported lumbopelvic pain (PGP and/or LBP) at 12 to 32 weeks of gestation (n=60) | LBP: provocation tests used: pain with lumbar flexion in standing, pain on palpation of paraspinal muscles, pain on trunk circumduction. PGP provocation test: P4 test | Weekly yoga sessions | Pamphlet advising on postural orientation in daily activities |
| McIntyre et al (1996) | Australia | Intervention study | Pregnant women reporting low back or pelvic pain during second or third trimester. N=20 | SIJ dysfunction determined by three tests; overtake phenomenon, fade test, Faber. Or pain reproduced on palpation over iliolumbar ligaments bilaterally | Rotational mobilisation treatment | No control |
| Miquelutti et al (2013) | Brazil | RCT | Pregnant women with a single foetus, age 16 to 40 years and gestational age 18 to 24 weeks, with or without lumbopelvic pain. (n=205) | Not defined | Physical and educational activities of the Birth Preparation Program conducted in addition to routine activities offered at the prenatal clinic. | Educational activities routinely offered at the prenatal clinics where they received prenatal care. |
| Mirmolae et al (2018) | Iran | RCT | Pregnant women 18 to 35 years old, were in the gestational week between 17 and 22, and had singleton pregnancy. (n=171) | Lumbopelvic pain - not differentiated | 12-week exercise program | Routine care |
| Mohamed et al (2018) | Egypt | RCT | Primiparous women, age 18-35, normal vaginal delivery, without epidural anaesthesia, breast feeding, BMI <30kg/m2, house wives, moderate education level, LBP complaints (n=30) | LBP anywhere from t12 to gluteal fold with or without radiation to the knee, of any intensity. | Exercise program 3 times a week for 2 weeks, instructions re back care, + kinesiotape for low back pain (3 applications for 3 days each) | Exercise program 3 times a week for 2 weeks, instructions re back care |
| Morkvad et al (2007) | Norway | RCT | Nulliparous women, 18 years or more, with a singleton live foetus at a routine ultrasound scan at 18 weeks of pregnancy. (n=301) | Pain in region of the pubic symphysis, over the sacroiliac joint area(s) (sometimes with radiation to the thighs), and in the lumbar region with or without radiation into one or both legs. | Specially designed exercise course including PFM and additional exercises, 12 weeks. | Customary information given by their midwife or general practitioner. |
| Murphy et al (2009) | USA | Prospective observational cohort | Pregnant women with LBP, Posterior pelvic pain, or both, onset after pregnancy, >18years (n=115) | Lumbopelvic pain; PPP, LBP or both | Management strategy that was founded on a diagnosis-based clinical decision rule | No control group |
| Noren et al (1997) | Sweden | Prospective, consecutive controlled cohort study | All pregnant women with any type of back pain, but subdivided into groups: PGP, lumbar pain, combined, nerve root syndrome. (n=135) | Posterior pelvic pain defined: pain drawings with markings distal and lateral to L5, positive P4 test, free ROM at hips and spine, pain-free intervals, history of time- and weight-bearing-related pain deep in gluteal area. | Individually designed program based on clinical assessment: education, exercises, taught not to overload the pelvis. Non-elastic support belt given. | Standard care |
| Oh et al (2007) | South Korea | RCT | Women between 36 and 39 weeks of gestation period, aged between 20 and 35 years, and reporting low back pain during pregnancy. (n=52) | Lumbar back pain, posterior pelvic pain, and mixtures of the two. | Back pain relief program: 8 weeks of exercise and education | No treatment |
| Ostgaard et al (1994) | Sweden | RCT | Pregnant women with back or pelvic pain (n=362) | Pelvic pain syndrome defined as all of the following: history of time- and weight-bearing pain in posterior pelvis, deep in gluteal region, pain drawing with markings of stabbing in buttocks distal and lateral to L5-S1, positive P4 test, free ROM hips and spine, no nerve root syndrome, pain on turning in bed. | (1) Group education classes - back school modified for pregnant women (two 45 min classes before 20th week pregnancy) with or without sacro-iliac belt.  (2) Individual education sessions (five 30 min sessions) with or without sacroiliac belt. | No intervention, routine ante-natal care |
| Ozdemir et al (2015) | Turkey | RCT | Pregnant women with pregnancy-related low back and/or pelvic pain, onset during pregnancy, >18years, 20-35 weeks gestation (n=96). At time of recruitment were doing < 30 mins exercise 3 days a week and not taking analgesics | Diagnosed by doctor with Low back and/or pelvic pain (LBPP) | Counselling and exercise program | Usual care |
| Pennick and Liddle (2013) | UK | Systematic review | Pregnant women at risk of or suffering from pelvic or back pain, as reported symptomatically or diagnosed by clinicians using specific tests. | Pelvic pain: (posterior pain arising from the region of the sacroiliac joints, anterior pain from the pubic symphysis, or both) | Various interventions: exercise (land- or water-based), pelvic belts, osteopathic manipulation (OMT), spinal manipulation (SMT) and neuro emotional techniques, a special pillow, acupuncture, acupuncture plus exercises, and a multi-modal approach incorporating manual therapy, exercise and education | Usual prenatal care (in some trials referred to as ’no treatment’), or usual prenatal care plus another intervention |
| Pennick and Young (2007) | Canada | Systematic review | Pregnant women suffering from or at risk of back pain or pelvic pain (n= 1305) | As defined by study | Various interventions added to usual prenatal care (exercises, water exercises, special pillow, acupuncture) | Either no treatment added to usual care, or another treatment type added to usual care |
| Peterson et al (2012) | USA | RCT | Pregnant women with a singleton and had low back pain of unknown origin that began during pregnancy and was reproducible by manual palpation. (n=57) | Pain in lumbar or sacroiliac region | (1) Exercise program (booklet + instructions)  (2) Spinal manipulative therapy  (3) Neuro emotional technique | No control |
| Peterson et al (2014) | Swizerland | Prospective cohort outcomes study | Pregnant patients age> 18 with low back pain, pelvic pain, or both of any duration who have not undergone chiropractic or manual therapy in the prior 3 months (n=115) | LBP, PGP, or both. Self-report. | Chiropractic treatment (specific methods/frequencies at discretion of treating clinician) | No control |
| Richards et al (2012) | Australia | Systematic review | Pregnant women of any gestational age, with any low back and/or pelvic pain (n=566) | Not defined | Acupuncture, exercise and abdominal/pelvic belts | No treatment/usual care or another treatment type |
| Schiff et al (2012) | USA | Systematic review | Pregnant women with or without LBP and/or PGP, in second or third trimester of pregnancy. | Not defined | Exercise interventions | Not specified |
| Schwerla et al (2015) | Germany | RCT | Women aged between 18 and 42 years, delivered a child within the past 3 to 15 months, and had at least 3 months of nonspecific LBP or PGP diagnosed according to the European guidelines. (n=80) | Diagnosed according to European guidelines and ≥5 on 10 point VAS for pain intensity | Osteopathic manipulation therapy 4 sessions, 40-60 minutes each | Waiting list and no other treatment allowed |
| Sedaghati et al (2007) | Iran | RCT | Pregnant women referred to prenatal clinics with or without LPP | Low back pain, defined as lumbar back pain, posterior pelvic pain, and a combination of the two. | Exercise program | No intervention |
| Sehmbi et al (2017) | Canada | Systematic review | Pregnant subjects with new onset or worsening of pre-existing LBP of moderate-to-severe intensity (numerical rating score for pain of 4 or more on a 0–10 scale) | Pregnancy-related LBP (between 12th rib and gluteal fold) including pregnancy-related PGP (between posterior iliac crest and gluteal fold) | Multimodal Management Including Patient Education, Exercise, and Psychological Therapies; physical therapy and exercise; Complementary and Alternative Medicine; non-surgical interventions; surgical interventions | Not specified |
| Shim et al (2007) | South Korea | Control group study | Pregnant women between 17 and 22 weeks of pregnancy, aged between 20 and 35 years, who reported lumbar and/ or posterior pelvic pain during pregnancy.(n=56) | LBP and/or posterior pelvic pain or both. Self-report. | 12-week back pain reducing program; pamphlet, lecture, audio-visual tape to demonstrate the exercise, daily exercise record, and telephone calls. | No intervention |
| Shiri et al (2018) | Finland/UK | Systematic review | Pregnant women with and without LBP or PGP, where at least some of the women have no pain at baseline (n=2347) | Self-report of symptoms | Exercise interventions | Not specified |
| Sklempe et al (2017) | Croatia | RCT | Healthy pregnant women or women diagnosed with mild gestational diabetes mellitus treated only by diet and lifestyle change, with no other medical conditions, age 20- 40, upper limit 30 weeks gestation at beginning of treatment. (n=42) | Lumbopelvic pain - not differentiated | Supervised, individualized therapeutic exercise programme consisting of aerobic and resistance exercises along with daily vigorous walks | Standard antenatal care, not discouraged from exercising on their own |
| Stafne et al (2012) + Stafne (2011) duplicate/ conference abstract | Norway | RCT | Pregnant women age ≥18, with singleton live foetus. (n=761) | LBP and PGP no differentiation. Self-report 'do you have pain in the lumbar and/or pelvic area?' | 12 week regular exercise program. Sessions of 60 min in groups of 8–15 women instructed by a physiotherapist were offered once a week over a period of 12 weeks (between 20 and 36 weeks of pregnancy), and written 45 min home exercise program at least twice a week | Standard antenatal care, not discouraged from exercise |
| Stuge et al (2003) | Norway | Systematic review | Pregnant women or women in the postpartum period (within 1 year after giving birth), with or without pelvic pain or low back pain. (n=1350) | Pregnancy-related low back or pelvic pain | Physical therapy exercise interventions including exercise, back school, massage, mobilization, electrotherapy, use of a sacroiliac belt and water gymnastics. | Not specified |
| Ternov et al (2001) | Sweden | Retrospective intervention study | Pregnant patients without a history of infertility or more than one spontaneous abortion with lower back pain, pelvic pain or both (n=167) | Not defined | Acupuncture | No control |
| Tseng et al (2015) | UK | Systematic review | Postnatal women who reported LPP onset either in pregnancy or within 3 months after delivery. (n=251) | Not defined | Physical therapy with a suite of exercise programs specifically designed to strengthen deep local muscles and global muscles in the lumbopelvic region | No therapy; or physical therapy using other methods such as massage relaxation, joint mobilization, manipulation, electrotherapy, hot packs, and simple back strengthening exercises |
| Van Benton et al (2014) | The Netherlands | Systematic review | Pregnant women with or without lumbopelvic pain (LBP, PGP or both) | Inclusion criteria differed between studies (self-report, pain on palpation) | All non-pharmacological interventions performed by physical and manual therapists, osteopaths, or chiropractors 4 categories: a combination of interventions (often with educational programs), exercise therapy, manual therapy, and material support. | Not specified |
| Van Kampen et al (2015) | Belgium | Systematic review | Primiparous, and multiparous women during pregnancy, receiving prenatal physiotherapy to prevent and/or reduce symptoms | LBP, or PGP, or both | Prenatal physiotherapy | Not specified |
| Van Zwienen et al (2004) | The Netherlands | Single-group prospective follow-up study | Persistent pregnancy-related low back and pelvic pain, where patient had completed all conservative treatment options, including a multidisciplinary rehabilitation program, and showed no significant improvement. Last delivery at least 12 months prior to inclusion (n=58) | Diagnosis: pain in one or both SIJs that originated during the pregnancy or directly after the delivery and increased during ADL activities and exercise. Both provocation tests (ASLR and P4) had to be positive. Additionally, radiological evidence required and severe disability in mobility and self-care (Barthel index <20) | Surgical procedure: SIJ fixation | No control |
| Vas et al (2014) | Spain | RCT (4-armed) (protocol) | Pregnant women  (24 to 36 weeks’ gestation), aged >17, referred by their family physicians diagnosed with pregnancy-related LBPGP, who have not previously received auricular acupuncture. (n=212) | LBP, PGP, both, +/- radiating to leg | Auricular acupuncture with pressure needles inserted in the ear (VAAc) together with Standard obstetric care (SOC) | SOC alone |
| Wang et al (2009) | USA | RCT (pilot study) | Pregnant women with a gestational age of 25-38 weeks, who had lower back and/or posterior pelvic pain. All participants were healthy women with a physical status II in the American Society of Anaesthesiologists (ASA) classification and had no prior experience with acupuncture | PRLP was classified based on the classification defined by Norén et al and exclusion criteria included any associated nerve root syndrome, neurologic deficit, fever, abdominal pain, other systemic manifestations, and active uterine contractions. | (1) Acupuncture at specific auricular points  (2) Acupuncture at nonspecific auricular points. | No intervention |
| Wedenberg et al (2000) | Sweden | RCT | Pregnant women no more than 32 weeks gestation, suffering from low back and pelvic pain (n=60) | Diagnosis by physiotherapist, based on patient history, a ‘pain-drawing’ by the patient indicating the location and type of pain, examination of the back and pelvis, and the results of provocation tests for pain originating from the pelvis and for pelvic instability | Acupuncture; 10 sessions of 30 minutes in a month | Physiotherapy; 10 treatments within 6-8 weeks, 50 minutes each session |
| Wiesner et al (2017) (Conference abstract) | Germany | RCT | Women with pregnancy-related low back pain, between 30th and 36th of pregnancy. (n=40) | Not specified | Osteopathic + additional intravaginal treatment | Osteopathic treatment only |
| Yao et al (2017) | China | Systematic review and meta-analysis | Women who were undergoing pregnancy or postpartum period and simultaneously suffering from LBP and/or PGP.  (n=1094) |  | Acupuncture treatment | Conventional therapy including exercise, herb, local injection and supportive care |
| Young et al (2002) | UK | Systematic reviews | Any pregnant women suffering from or at risk of LBP and/or PGP | LBP and/or PGP | Any intervention aimed at treating LBP or PGP; special pillow, aquatic exercise, acupuncture and physiotherapy | Not specified |
