# Supplementary material for: Outcomes and outcomes measurements used in intervention studies of pelvic girdle pain and lumbopelvic pain: a systematic review
Source: Chiropr Man Therap. 2019 Nov 5;27:62. doi: 10.1186/s12998-019-0279-2 (PMC6829811; doi:10.1186/s12998-019-0279-2)
Supplement: Supplementary file 3 — Additional file 3. Quality of reporting. The results of the assessment of the quality of reporting in the individual studies included in this systematic review. [file 12998_2019_279_MOESM3_ESM.docx]

**Additional file 3: Quality of reporting**

1. Quality of reporting questions for studies on PGP:

| Author/year | Q.1 | Q.2 | Q.3 | Q.4 | Q.5 | Q.6 |
| --- | --- | --- | --- | --- | --- | --- |
| Almousa et al (2018) | yes | yes | N/A | N/A | no | N/A |
| Almousa et al (2015) | yes | no | no | no | no | NA |
| Barfoot et al (2015) (Conference abstract) | yes | yes | NA | NA | no | yes |
| Bertuit et al (2018) | yes | yes | NA | NA | yes | yes |
| Bhandiwad et al (2015) (Conference abstract) | yes | yes | NA | NA | no | no |
| Bromley and Bagley 2014 | yes | yes | NA | NA | yes | NA |
| Cameron et al (2017) (Conference abstract) | yes | yes | NA | NA | no | yes |
| Cameron et al (2015) (Conference abstract) | yes | yes | yes | yes | no | yes |
| Clarkson et al (2016) (Conference abstract) | yes | yes | NA | NA | no | no |
| Depledge et al (2005) | yes | yes | N/A | N/A | yes | yes |
| Elden et al (2008)(a) | yes | yes | yes | yes | yes | yes |
| Elden et al. (2008)(b) | yes | yes | NA | NA | yes | yes |
| Elden et al (2005) | yes | yes | yes | yes | yes | yes |
| Elden et al (2008)(c) | yes | yes | NA | NA | yes | yes |
| Elden et al (2013) | yes | yes | yes | yes | yes | yes |
| Flack et al (2015) | yes | yes | yes | yes | yes | yes |
| Gausel et al (2017) | yes | yes | yes | yes | yes | yes |
| Gupta et al (2015) (Conference abstract) | yes | yes | NA | NA | no | yes |
| Haugland et al (2006) | yes | yes | yes | yes | yes | yes |
| Kibsgard et al (2014) | yes | yes | yes | yes | yes | yes |
| Kuciel et al (2017) | yes | yes | NA | NA | yes | yes |
| Ladfors et al (2004) | yes | yes | NA | NA | no | yes |
| Lund et al (2006) | yes | yes | NA | NA | yes | yes |
| Melkersson et al (2017) | yes | yes | NA | NA | yes | yes |
| Mens et al (2000) | yes | yes | yes | yes | yes | yes |
| Nilsson-Wikmar et al (2005) | yes | yes | NA | NA | yes | yes |
| Ribnikar et al (2015) (Conference abstract) | yes | yes | NA | NA | no | no |
| Schep et al (2004) | yes | yes | NA | NA | yes | yes |
| Stuge et al (2004)+ Stuge et al (2004) 2 year follow up. | yes | yes | yes | yes | yes | yes |
| Torstensson et al (2013) | yes | yes | NA | NA | yes | yes |
| Torstensson et al (2009) | yes | yes | yes | yes | yes | yes |
| Vaidya (2018) | yes | yes | NA | NA | yes | yes |
| Weil et al (2008) | yes | yes | NA | NA | yes | yes |

(ii) Quality of reporting questions for studies on LBPP:

| Author/ year | Q.1 | Q.2 | Q.3 | Q.4 | Q.5 | Q.6 |
| --- | --- | --- | --- | --- | --- | --- |
| Abu et al (2017) | yes | yes | NA | NA | yes | yes |
| Al-Sayegh et al (2010) | yes | yes | yes | yes | yes | yes |
| Balasundaram et al. (2017) | no | no | no | no | no | no |
| Barkatsa et al (2010) (Conference abstract) | yes | no | no | no | no | no |
| Bastiaenen et al (2006) | yes | yes | yes | yes | yes | yes |
| Bastiaenen et al (2008) (Long term follow up of Bastiaenen et al (2006)) | yes | yes | yes | yes | yes | yes |
| Bastiaenen et al (2004) *protocol for 2006 paper | yes | yes | yes | yes | yes | yes |
| Bennett (2014) | yes | yes | NA | NA | yes | NA |
| Bishop et al (2016) + Foster et al (2016) | yes | yes | NA | NA | yes | yes |
| Butel et al (2016) (Conference abstract) | yes | yes | yes | yes | yes | no |
| Close et al 2014 | yes | N/A | yes | N/A | no | NA |
| Close et al (2016) | yes | yes | yes | yes | yes | yes |
| Daly et al (1991) | yes | yes | NA | NA | yes | yes |
| Ee et al. (2008) | yes | N/A | N/A | N/A | no | N/A |
| Eggen et al (2012) | yes | yes | yes | yes | yes | yes |
| Ekdahl and Peterson (2010) | yes | yes | NA | NA | yes | yes |
| Ferreira and Alburquerque-Sendin (2013) | no | no | N/A | N/A | no | N/A |
| Fisseha and Mishra (2016) | yes | yes | NA | NA | yes | NA |
| Franke et al (2017) | yes | yes | yes | yes | yes | NA |
| George et al (2013) | yes | yes | yes | yes | yes | yes |
| Granath et al (2006) | yes | yes | NA | NA | yes | yes |
| Gross et al (2012) (Conference abstract) | yes | yes | NA | NA | yes | yes |
| Guerreiro da Silva et al (2004) | yes | yes | yes | yes | yes | yes |
| Gutke et al 2010 (+Gutke 2011 abstract) | yes | yes | yes | yes | yes | yes |
| Gutke et al (2015) | NA | NA | NA | NA | yes | NA |
| Hall et al (2016) | yes | yes | yes | yes | yes | NA |
| Haakstad and Kari (2015) | yes | yes | yes | yes | yes | yes |
| Hilde et al (2016) | yes | yes | yes | yes | yes | NA |
| Ho et al (2009) | no | NA | NA | NA | NA | NA |
| Kalus et al (2008) | yes | yes | yes | yes | yes | yes |
| Kaplan et al (2016) | yes | yes | NA | NA | yes | yes |
| Kinser et al (2017) | yes | yes | yes | yes | yes | NA |
| Kluge et al (2011) | yes | yes | yes | yes | yes | yes |
| Kordi et al (2013) | yes | yes | NA | NA | yes | yes |
| Kvorning et al (2004) | yes | yes | NA | NA | yes | yes |
| Liddle and Pennick (2015) | yes | yes | NA | NA | yes | yes |
| Lillios et al (2012) | NA | NA | NA | NA | no | NA |
| Majchrzycki et al (2015) | no | no | no | no | no | no |
| Martins and Silva (2014) | yes | yes | NA | NA | yes | yes |
| McIntyre et al (1996) | yes | yes | na | na | yes | no |
| Miquelutti et al (2013) | yes | yes | NA | NA | yes | yes |
| Mirmolae et al (2018) | yes | yes | NA | NA | yes | yes |
| Mohamed et al (2018) | yes | yes | na | na | yes | yes |
| Morkvad et al (2007) | yes | yes | NA | NA | yes | yes |
| Murphy et al (2009) | yes | yes | yes | yes | yes | yes |
| Noren et al (1997) | yes | yes | NA | NA | yes | yes |
| Oh et al (2007) | yes | yes | NA | NA | yes | yes |
| Ostgaard et al (1994) | yes | yes | NA | NA | yes | yes |
| Ozdemir et al (2015) | yes | yes | yes | yes | yes | yes |
| Pennick and Liddle (2013) | yes | yes | NA | NA | yes | yes |
| Pennick and Young (2007) | yes | yes | NA | NA | yes | NA |
| Peterson et al (2012) | yes | yes | yes | yes | yes | yes |
| Peterson et al (2014) | yes | yes | NA | NA | yes | yes |
| Richards et al (2012) | yes | yes | NA | NA | yes | NA |
| Schiff et al (2012) | yes | no | NA | NA | yes | NA |
| Schwerla et al (2015) | yes | yes | yes | yes | yes | no |
| Sedaghati et al (2007) | yes | yes | NA | NA | yes | yes |
| Sehmbi et al (2017) | yes | yes | NA | NA | yes | NA |
| Shim et al (2007) | yes | yes | NA | NA | yes | yes |
| Shiri et al (2018) | yes | yes | NA | NA | yes | NA |
| Sklempe et al (2017) | yes | yes | NA | NA | yes | yes |
| Stafne et al (2012) + Stafne (2011) duplicate/ conference abstract | yes | yes | yes | yes | yes | yes |
| Stuge et al (2003) | yes | no | NA | NA | no | NA |
| Ternov et al (2001) | yes | yes | NA | NA | yes | no |
| Tseng et al (2015 | yes | yes | NA | NA | yes | NA |
| Van Benton et al (2014) | yes | yes | NA | NA | yes | NA |
| Van Kampen et al (2015) | yes | no | NA | NA | yes | NA |
| Van Zwienen et al (2004) | yes | yes | NA | NA | yes | yes |
| Vas et al (2014) | yes | yes | yes | yes | yes | yes |
| Wang et al (2009) | yes | yes | NA | NA | yes | yes |
| Wedenberg et al (2000) | yes | yes | NA | NA | yes | yes |
| Wiesner et al (2017) (Conference abstract) | yes | yes | yes | yes | no | yes |
| Yao et al (2017) | yes | yes | NA | NA | yes | NA |
| Young et al (2002) | yes | yes | NA | NA | yes | NA |
