# Supplementary material for: Outcomes and outcomes measurements used in intervention studies of pelvic girdle pain and lumbopelvic pain: a systematic review
Source: Chiropr Man Therap. 2019 Nov 5;27:62. doi: 10.1186/s12998-019-0279-2 (PMC6829811; doi:10.1186/s12998-019-0279-2)
Supplement: Supplementary file 4 — Additional file 4. Comparison of outcomes identified in PGP and LPP studies for each core domain. The outcomes that were identified in studies examining PGP only are compared to the outcomes identified in studies including patients with LPP. This comparison has been presented by core domain. [file 12998_2019_279_MOESM4_ESM.docx]

**Additional file 4: Comparison of outcomes identified in PGP and LPP studies for each core domain**

| Domain | PGP | LPP |
| --- | --- | --- |
| Life Impact |  |  |
| *Pain outcomes* | Pain intensity | Pain intensity |
|  | Pain location | Pain location |
|  |  | Pain frequency |
|  |  | Pain prevalence |
|  |  | Pain behaviour |
| *Functional outcomes* | Function | Function |
|  | Functional mobility | Functional mobility |
|  | Physical activity levels |  |
|  | Disability | Disability |
|  |  | Work disability |
| *QOL/ Health status* | HR-QOL | HR-QOL |
|  | Health status | Health status |
|  | Perceived health |  |
| *Other* | Perceived improvement | Perceived improvement |
|  | Patient satisfaction (with treatment/ life) | Patient satisfaction (with treatment/ life) |
|  |  | Patient expectations of treatment |
| *Psychological outcomes* | confidence | Fear avoidance/ fear of movement |
|  | Self-efficacy | Pain catastrophizing |
|  |  | General mental health |
|  |  | Anxiety |
|  |  | Wellbeing |
|  |  | depression |
| *Sleep/ fatigue* | Fatigue (morning and evening) | Trouble sleeping |
| Resource-use/ economic impact |  |  |
|  | Sick leave/ temporary occupational incapacity | Sick leave/ temporary occupational incapacity |
|  | Analgesia use | Analgesia use |
|  |  | Cost |
|  |  | Work performance |
|  |  | Healthcare utilisation |
| Pathophysiological manifestations |  |  |
|  | Pain location/ pain provocation | Pain location/ pain provocation |
|  | Recovery of symptoms |  |
|  | posture | posture |
|  | continence | continence |
|  | Pubis symphysis mobility |  |
|  | Muscle function (strength/ endurance) | Muscle function (strength/ endurance) |
|  | Gait speed/endurance | Gait speed/endurance |
|  |  | Flexibility |
|  |  | Anthropometric outcomes |
|  | Pregnancy outcomes / maternal outcomes | Pregnancy outcomes / maternal outcomes |
|  | Surgical outcomes(fluoroscopy time, insertion time for guide wires, operation time, screw position) |  |
| Adverse Events |  |  |
|  | Adverse events (not specified) | Adverse events (not specified) |
|  | Post-op complications | Fetal outcome |
|  |  | Safety of women and children |
